# ICTV Virus Taxonomy Profile: *Benyviridae*

**DOI:** 10.1099/jgv.0.000864

**Published:** 2017-07-17

**Authors:** David Gilmer, Claudio Ratti, ICTV Report Consortium

**Affiliations:** ^1^​ Integrative Virology, Université de Strasbourg, CNRS, IBMP UPR 2357, F-67000 Strasbourg, France; ^2^​ Università di Bologna, DipSA, 40127 Bologna, Italy

**Keywords:** *Benyviridae*, ICTV Report, beet necrotic yellow vein virus

## Abstract

The *Benyviridae* is a family of multipartite plant viruses with rod-shaped virions. Genomes are segmented and comprised of single-stranded, positive-sense RNAs, each with a 5′ m^7^G cap. Unlike rod-shaped viruses classified in the *Virgaviridae* family, the genome segments have a 3′ polyA tract and there is post-translational cleavage of the viral replicase. The better-known members are transmitted by root-infecting vectors in the Plasmodiphorales family, once described as fungi but now classified as Cercozoa. The family has a single genus. This is a summary of the International Committee on Taxonomy of Viruses (ICTV) Report on the taxonomy of *Benyviridae,* which is available at www.ictv.global/report/benyviridae.

## Virion

**Fig. 1.**
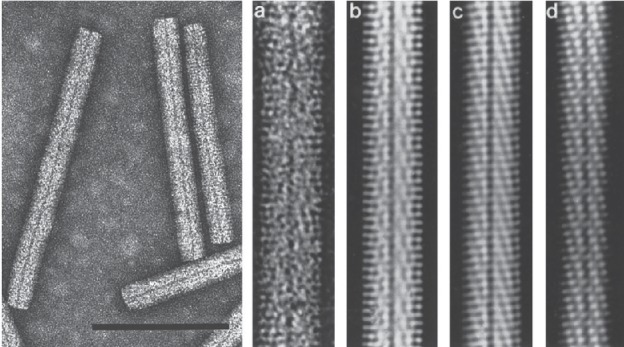
Electron micrographs of beet necrotic yellow vein virus particles. (Left) Negative contrast-stained purified particles. (Right) From the left, (a) negative contrast electron micrograph and (b, c, d) computer-filtered micrographs (modified from [1], courtesy of A.C. Steven). The bar represents 100 nm.

Non-enveloped, helically constructed rod-shaped particles, with an axial canal and up to five different lengths have been described (Table 1 and Fig. 1). In beet necrotic yellow vein virus, the predominant lengths are about 390, 265, 100, 85 and 65–80 nm and their diameter is about 20 nm. The right-handed helix with a pitch of 2.6 nm has an axial repeat of four turns, involving 49 coat protein subunits, each occupying four nucleotides [1].

**Table 1.** Characteristics of the *Benyviridae* family

**Typical member:**	beet necrotic yellow vein virus isolate Japan S (RNA1: D84410; RNA2: D84411; RNA3: D84412; RNA4: D84413; RNA5: D63936), species *Beet necrotic yellow vein virus*, genus *Benyvirus*
Virion	Non-enveloped, rod-shaped particles about 20 nm in diameter and up to about 390 nm long with two or more modal lengths
Genome	Two segments of polyadenylated positive-sense RNA (approximately 7 and 4.6 kb) and up to three additional RNA components of 1.3–1.8 kb
Replication	Cytoplasmic, probably associated with the endoplasmic reticulum
Translation	Directly from genomic or intracellular subgenomic RNAs
Host range	Plants
Taxonomy	One genus

## Genome

Beet necrotic yellow vein virus has four or five linear positive-sense ssRNAs of about 6.7, 4.6, 1.8, 1.4 and 1.3 kb, respectively [2, 3]. The viral RNAs are capped at the 5′ end and 3′-polyadenylated, unlike the RNAs of all other rod-shaped plant viruses. Viral RNAs have a conserved 3′ structure involved with RNA replication initiation. After mechanical transmission to laboratory test plants, RNA3, RNA4 and RNA5 may carry deletions or be lost entirely [4]. Isolates of the beet soil-borne mosaic virus have four RNAs [5], while rice stripe necrosis virus [6] and burdock mottle virus [7] apparently only contain two genomic RNAs.

## Replication

**Fig. 2.**
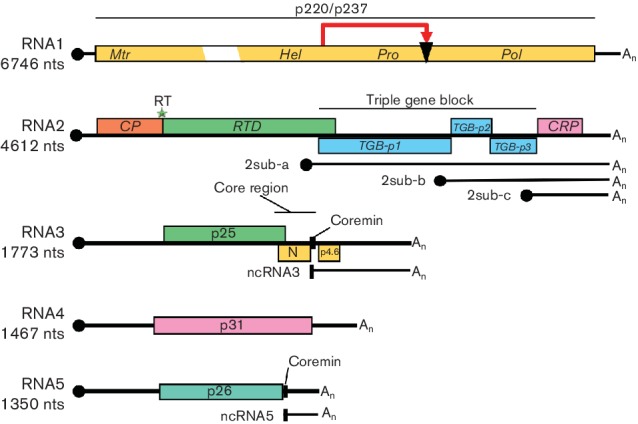
Genome organization and translation strategy of beet necrotic yellow vein virus. The scheme indicates self-cleavage of the replicase protein (red arrow and black triangle), a suppressible UAG stop codon (green star), m^7^Gppp (black circle) and the 3′ poly(A) tails (An). Mtr, methyltransferase; Hel, helicase; Pro, protease; Pol, RNA polymerase; RT, readthrough; RTD, readthrough domain; sub, subgenomic; CRP, cysteine rich protein. N and p4.6 have never been detected. Noncoding RNAs, ncRNA3 and ncRNA5, produced by exoribonuclease activity harbour the conserved coremin motif present in the ‘core region’ responsible for long distance movement in *Beta* species.

RNA1 contains one large open reading frame (ORF) coding for a replication-associated protein that is cleaved post-translationally (Fig. 2). This proteolytic cleavage distinguishes benyviruses from all other viruses with rod-shaped particles, which have their replication-associated proteins encoded in two ORFs. The protein contains motifs for methyltransferase, helicase, a papain-like protease and an RNA-dependent RNA polymerase. RNA2 contains six ORFs, the first of which encodes the major coat protein of 21–23 kDa and is terminated by an amber stop codon (UAG). When this codon is suppressed, the minor coat protein readthrough protein is translated: this contains a KTER motif in its C terminal part that is necessary for the transmission of the virions by *Polymyxa betae* [8].

Downstream of the coat protein ORF are three genes of the ‘triple gene block’ encoding proteins of 42, 13 and 15 kDa, respectively that are associated with cell-to-cell movement. At a 3′ proximal position, RNA2 contains a gene encoding a 14 kDa cysteine-rich protein that is a suppressor of post-transcriptional gene-silencing [9]. The downstream ORFs described above are expressed by the subgenomic mRNAs, 2sub-a, 2sub-b and 2sub-c. RNAs 1 and 2 are sufficient for replication of beet necrotic yellow vein virus in experimental hosts but the typical rhizomania symptoms in beet are produced only in the presence of RNA3; RNA4 greatly increases the transmission rate by *Polymyxa betae* and RNA5 may modulate the type of symptoms formed. RNA3 and RNA5 lead to accumulation of noncoding RNA3 and non coding RNA5 [10]. RNA3 and RNA4 are always present in natural beet necrotic yellow vein virus infections. Beet necrotic yellow vein virus is able to replicate and encapsidate beet soil-borne mosaic virus RNA3 and RNA4. Genome RNA amplification occurs in the cytoplasm from complementary-strand intermediate(s).

## Taxonomy

There is a single genus, *Benyvirus*. Beet necrotic yellow vein virus causes the widespread (Europe, North America, Asia) and highly damaging soil-borne ‘rhizomania’ disease of sugar beet.

## Resources

Full ICTV Online (10th) Report: www.ictv.global/report/benyviridae.
